# Identifying factors associated with central obesity in school students using artificial intelligence techniques

**DOI:** 10.3389/fped.2022.1060270

**Published:** 2022-11-30

**Authors:** Yicheng Zhang, Qiong Wang, Mei Xue, Bo Pang, Min Yang, Zhixin Zhang, Wenquan Niu

**Affiliations:** ^1^ Graduate School, Beijing University of Chinese Medicine, Beijing, China; ^2^ Department of Pediatrics, China-Japan Friendship Hospital, Beijing, China; ^3^ International Medical Services, China-Japan Friendship Hospital, Beijing, China; ^4^ Institute of Clinical Medical Sciences, China-Japan Friendship Hospital, Beijing, China

**Keywords:** central obesity, school students, artificial intelligence, risk factors, prediction

## Abstract

**Objectives:**

We, in a large survey of school students from Beijing, aimed to identify the minimal number of promising factors associated with central obesity and the optimal machine-learning algorithm.

**Methods:**

Using a cluster sampling strategy, this cross-sectional survey was conducted in Beijing in early 2022 among students 6–14 years of age. Information was gleaned *via* online questionnaires and analyzed by the PyCharm and Python.

**Results:**

Data from 11,308 children were abstracted for analysis, and 3,970 of children had central obesity. Light gradient boosting machine (LGBM) outperformed the other 10 models. The accuracy, precision, recall, F1 score, area under the receiver operating characteristic of LGBM were 0.769982, 0.688312, 0.612323, 0.648098, and 0.825352, respectively. After a comprehensive evaluation, the minimal set involving top 6 important variables that can predict central obesity with descent performance was ascertained, including father's body mass index (BMI), mother's BMI, picky for foods, outdoor activity, screen, and sex. Validation using the deep-learning model indicated that prediction performance between variables in the minimal set and in the whole set was comparable.

**Conclusions:**

We have identified and validated a minimal set of six important factors that can decently predict the risk of central obesity when using the optimal LGBM model relative to the whole set.

## Introduction

Childhood obesity is a global problem and it is increasing to epidemic proportions (1, 2). As reported by the Global Burden of Disease Study 2013, the prevalence of obesity in children and adolescents has substantially increased around the world, especially in developing countries, from 8.1% to 12.9% for boys and from 8.4% to 13.4% in girls in 2013 (3). In China, the prevalence of overweight or obesity was 5.3% in 1995, and this number was skyrocketed to 20.5% in 2010 (4). Given the facts that obesity in childhood frequently persists into adulthood and obesity is an established risk factor for many chronic diseases (5), a better understanding of the etiology of childhood obesity can facilitate the development of effective strategies for preventing this outcome and its resultant sequelae.

It is well known that obesity is a complex, multifactorial disease with a highly inheritable tendency. There is evidence that children who have parents/grandparents with obesity are at higher risk of becoming obese than others. Besides, lifestyle-related factors such as eating habits and sleep duration also play a contributory role in the development of childhood obesity. In the literature, the majority of studies have examined risk profiles of childhood obesity using body mass index, which is a reflection of general obesity. As compared with general obesity, central obesity is a strong risk factor for cardio-metabolic disorders in children and adolescents (6, 7) and their unfavorable prognoses (8–11), because the endocrine of abdominal fat is more vigorous (12). In an observational study, central obesity in children who were school-aged was found to be associated with the development of autoimmune diseases, but being overweight was not (13). To this point, it is important to determine the risk factors behind central obesity in children. In a large sample of children who were school-aged from Greece, frequent breakfast, snack consumption, and frequent participation in sedentary activities were the strongest lifestyle determinants of central obesity (14). Another study indicated that higher adherence to the Mediterranean dietary pattern and higher cardiorespiratory fitness were associated with lower waist circumference in preschool children (15). Considering the complex etiology of central obesity, delineation of potential nonlinear, collinear or synergistic contributions of individual risk factors is challenging and beyond the capability of traditional statistical methods, like Logistic regression analysis. Fortunately, advancements in machine-learning and deep-learning techniques can at least partly shed some light on this challenge (16), due to their versatility, power and scalability in solving large and highly complex tasks.

To produce more information, we decided to survey factors from both students and parents and employ machine-learning techniques, aiming to identify the minimal number of promising factors associated with central obesity and the optimal machine-learning algorithm with decent performance, which can be applied in practical settings to predict the risk of childhood central obesity.

## Materials and methods

### Study design and ethical approval

This survey is designed to cross-sectionally collect data from students and their parents. Students from 26 schools located in a suburban district (Ping Gu) of Beijing were surveyed during the first month of 2022. The implementation of this survey conformed to the principles in the Declaration of Helsinki, and was approved by the Ethics Committee of Beijing University of Chinese Medicine.

### Study participants

Students aged 6–14 years from 8 primary schools and 18 junior schools in Ping Gu district formed the study participants. With the exception of severe endocrine disorders, including but not limiting to hyperthyroidism, hypothyroidism and diabetes mellitus, all students are deemed eligible for inclusion.

At first, this survey included 11,633 students whose parents or guardians were requested to complete the questionnaire on smartphone. Finally, 11,308 questionnaires were valid, with a return rate of 98%.

### Data collection

Survey was deployed by means of self-designed questionnaire. This questionnaire is sent electronically to the parents or guardians of students who attended primary schools or junior schools in the form of QR code by their class teachers. The class teachers and school health physicians were trained online about how to understand and fill in the questionnaire.

This questionnaire was designed on a network platform named “Wenjuanxing” (available at https://www.wenjuan.com/). At the end of survey, data were downloaded as an Excel file from this platform and were checked by research scientists.

### Quality control

Before circulating this questionnaire, reliability coefficient (alpha) was calculated a prior and it exceeded 0.85. As data from this survey were collected online, it is essential to ensure the quality. All data were double checked by trained staff. In case of missing data or data with extreme values, school class teachers were contacted by re-inviting the parents or guardians to provide or validate data.

### Definition of central obesity

As students in this study are in growth periods, height-dependent central obesity is preferred for practical applications. To this point, the cut-off value of waist-to-height ratio (WHtR) is used to define central obesity, and this value is referenced based on age and sex. In the present study, the cut-off value of WHtR is set at 0.46 for girls and 0.48 for boys according to previous reports (17, 18).

### Items in questionnaire

Items in the questionnaire were designed to cover information from both students and their parents. Information from students covered birth date, sex, gestational age (in weeks), pregnant and birth order, delivery mode (natural labor or cesarean section), twins (yes or no), birth weight (in grams), birth height (in centimeters), breastfeeding duration (in months), solid food introduction age (in months), weight (in kilograms), height (in centimeters), hip and waist circumference (in centimeters), chronic diseases, family history of diabetes and hypertension, lifestyle habits such as mean daily outdoor duration (in hours), mean daily sitting time (in hours), mean daily screen time (in hours), fall asleep time and sleep duration (in hours), eating habits (fussy eating or not, frequency of snacks and other food intakes), and stool customs (frequency and character). Height and weight were measured by school health physicians.

Information from parents included age, body height (self-reported in centimeters), weight (self-reported in kilograms), education, and family annual income (RMB).

### Definition of items

Waist circumference was horizontally measured at about a centimeter above the navel, and hip circumference was at the most protruding point level of their hips. Medical history of students referred to chronic kidney disease, hypothyroidism, congenital heart disease, chronic respiratory diseases, and other chronic diseases diagnosed from second-class or above hospitals. Delivery modes included natural birth, c-section and forceps delivery. Pregnant order and delivery order were divided into 2 groups as <2 and ≥2. Fiber foods included fruits and vegetables in season and grains. Animal protein refers to meat and processed meat. Soy protein was to point to legume and bean product. Dietary supplements included tonics such as royal jelly. Fast food referred to foods that are high in energy and low in nutrition (such as hamburgers and French fries). Night meal was to point to eating within 2 h of bedtime. Sleep duration, duration of physical activity, and daily sitting time were separately calculated as the sum of both corresponding time on workdays × 5 and corresponding time on weekends × 2 divided by 7. Stool character was defined according to the Bristol Stool Scale (BSFS) (19), and it was divided into 4 categories: individual lumps like nuts; like sausages but lumpy; like sausages, but with cracks on the surface; like sausages and smooth and soft, fluffy, watery. Family history of diabetes or hypertension was expressed by the number of parents and grandparents who were clinically diagnosed with diabetes or hypertension. Education level of parents was divided into senior high school/technical secondary school and below, undergraduate/junior college, and graduate school or above. Family annual income was classified into <100,000, 100,000 to 300,000, and >300,000 RMB per year.

### Statistical analyses

After quality control, data were imported into the R programming environment (Version 4.1.1) for cleaning. Multiple-choice items were encoded as numbers. Missing data were imputed for multiple times (*N* = 5) with the R MICE package if percentage of missing values for each item is less than 30%, and were removed otherwise.

Continuous data were checked for normality, and if satisfied, they are expressed as mean (standard deviation) and median (quartile range) otherwise. Categorical data are uniformly expressed as number (percentage). Depending on the presence or absence of central obesity, data were divided into two groups. The distribution of survey items on either continuous or categorical scale were compared between the central obesity group and non-central obesity group by using *t*-test, rank-rum test or *χ*^2^ test where appropriate.

Machine-learning and deep-learning models are implemented using Integrated Development Environment (IDE) PyCharm Community Edition (2018.1 ×64) shipping the Python language (version 3.7.6). Models were trained on 60% of participating students (the training set) and tested on the remaining 40% (the validation set) as an internal validation of the central obesity-prediction model. In this study, 11 machine-learning models were respectively trained, including Logistic regression, random forest, support vector machine (SVM), decision tree, K-nearest neighbors (KNN), gradient boosting machine (GBM), light gradient boosting machine (LGBM), extreme gradient boosting machine (XGBoost), Gaussian naive Bayes (gNB), multinomial naive Bayes (mNB), and Bernoulli naive Bayes (bNB). Additionally, two decision-level fusion techniques, hard-voting and soft-voting classifiers, were applied based on above 11 machine-learning models. Model performance was assessed from five aspects, that is, accuracy (the prediction of correct outcomes as a percentage of the total sample), precision (the probability of the sample that was predicted to be positive being positive), recall score (the probability of being predicted to be a positive sample in a sample that is positive), F1 score (the harmonic mean of precision and recall), and AUROC (area under the receiver operating characteristic). The optimal model was selected after a comprehensive weighing up of the five aspects.

Generally, incorporation of more variables can improve model performance. For practical reasons, identification of a minimal set of variables that can capture much of model variation is critical. To achieve this goal, each variable was assigned an importance value generated by the *χ*^2^-based Scikit-learn feature selection method and the Shapley additive explanation (SHAP) tool, with a larger value corresponding to more importance in prediction for central obesity. Then, the importance of all variables under study was ranked in a descending order, and from the largest to the smallest, a panel of machine-learning models were generated by additional incorporation of one variable each time. The cumulative model performance was assessed by means of accuracy, precision, and AUROC, which were used to determine the minimal set of important variables.

Further, the prediction performance of variables in the minimal set as compared with the whole set was tested by the deep-learning sequential model, which was separately constructed with three different optimizers (adaptive moment estimation [Adam], root mean square prop [RMSprop], and stochastic gradient descent [SGD]). Model accuracy and model loss were computed for comparison in both training set and validation set.

## Results

### Baseline characteristics

Finally, data from 11,308 children were abstracted for analysis, and 3,970 of children (35.1%) had central obesity. Upon stratification by central obesity, the baseline characteristics of 11,308 students are shown in Table 1.

**Table 1:** The baseline characteristics of school students by the presence or absence of central obesity.

Factors under study	Non-central obesity (*n* = 7338)	Central obesity (*n* = 3970)	*P*
Baseline factors			
Sex (%)			<0.001
Boys	3,576 (48.7)	2,205 (55.5)	
Girls	3,762 (51.3)	1,765 (44.5)	
Age (months)	131.0[105.0,157.0]	66.4 [55.3, 72.7]	0.001
Lifestyle-related factors			
Frequency of picky for foods (%)			<0.001
None or occasionally	3,449 (47.0)	2,251 (56.7)	
1–2 times weekly	2,252 (30.7)	1,022 (25.7)	
3–5 times weekly	881 (12.0)	369 (9.3)	
Every day	756 (10.3)	328 (8.3)	
Intake frequency of out seasonable fruits (%)			0.021
None or occasionally	976 (13.3)	555 (14.0)	
1–2 times weekly	2,573 (35.1)	1,451 (36.5)	
3–5 times weekly	2,027 (27.6)	1,111 (28.0)	
Every day	1,762 (24.0)	853 (21.5)	
Intake frequency of animal proteins (%)			<0.001
None or occasionally	109 (1.5)	53 (1.3)	
1–2 times weekly	1,034 (14.1)	574 (14.5)	
3–5 times weekly	2,323 (31.7)	1,286 (32.4)	
Every day	3,872 (52.8)	2,057 (51.8)	
Intake frequency of snacks (%)			0.007
None or occasionally	1,404 (19.1)	864 (21.8)	
1–2 times weekly	4,121 (56.2)	2,143 (54.0)	
3–5 times weekly	1,263 (17.2)	688 (17.3)	
Every day	550 (7.5)	275 (6.9)	
Intake frequency of night meal (%)			0.005
None or occasionally	3,782 (51.5)	2,165 (54.5)	
1–2 times weekly	2,116 (28.8)	1,113 (28.0)	
3–5 times weekly	769 (10.5)	389 (9.8)	
Every day	671 (9.1)	303 (7.6)	
Intake frequency of sweet foods (%)			0.001
None or occasionally	1,397 (19.0)	842 (21.2)	
1–2 times weekly	4,198 (57.2)	2,288 (57.6)	
3–5 times weekly	1,293 (17.6)	647 (16.3)	
Every day	450 (6.1)	193 (4.9)	
Intake frequency of fast foods (%)			0.818
None or occasionally	3,321 (45.3)	1,762 (44.4)	
1–2 times weekly	3,473 (47.3)	1,910 (48.1)	
3–5 times weekly	342 (4.7)	191 (4.8)	
Every day	202 (2.8)	107 (2.7)	
Intake frequency of dietary supplements (%)			0.001
None or occasionally	6,003 (81.8)	3,358 (84.6)	
1–2 times weekly	717 (9.8)	351 (8.8)	
3–5 times weekly	268 (3.7)	122 (3.1)	
Every day	350 (4.8)	139 (3.5)	
Intake frequency of preservative foods (%)			0.094
None or occasionally	4,000 (54.5)	2,230 (56.2)	
1–2 times weekly	2,551 (34.8)	1,301 (32.8)	
3–5 times weekly	484 (6.6)	287 (7.2)	
Every day	303 (4.1)	152 (3.8)	
Frequency of sleeping in light (%)			0.646
None or occasionally	6,356 (86.6)	3,423 (86.2)	
1–2 times weekly	403 (5.5)	239 (6.0)	
3–5 times weekly	187 (2.5)	105 (2.6)	
Every day	392 (5.3)	203 (5.1)	
Stool consistency (%)			0.001
Separate hard lumps, like nuts	190 (2.6)	64 (1.6)	
Sausage-shaped but lumpy	1,004 (13.7)	524 (13.2)	
Like a sausage or snake but with cracks on its surface	1,382 (18.8)	692 (17.4)	
Like a sausage or snake smooth and soft, fluffy pieces, watery	4,762 (64.9)	2,690 (67.8)	
Stool frequency (%)			<0.001
1–2 times daily	5,349 (72.9)	3,048 (76.8)	
3–4 times daily	208 (2.8)	160 (4.0)	
>4 times daily	234 (3.2)	121 (3.0)	
2–3 times weekly	1,317 (17.9)	535 (13.5)	
0–1 times weekly	230 (3.1)	106 (2.7)	
Outdoor activities (hours per day)	1.29 [1.00, 1.71]	1.29 [1.00, 1.57]	<0.001
Sitting duration (hours per day)	1.29 [0.64, 1.64]	1.29 [0.86, 1.86]	<0.001
Electronic screens (hours per day)	1.00 [0.60, 1.60]	1.10 [0.60, 1.90]	<0.001
Sleep duration (hours per day)	9.00 [8.29, 9.29]	8.86 [8.29, 9.29]	0.032
Fall asleep time (post meridian)	10.00 [9.00, 10.00]	10.00 [9.00, 10.00]	0.084
Fetal and neonatal factors			
Bearing age of father (years)	30.60 [27.90, 34.20]	29.95 [27.22, 33.40]	<0.001
Bearing age of mother (years)	29.30 [27.00, 32.70]	28.90 [26.40, 32.00]	<0.001
Paternal BMI (kg/m^2^)	25.83 [23.44, 28.73]	26.73 [24.34, 29.86]	<0.001
Maternal BMI (kg/m^2^)	22.95 [20.80, 26.04]	24.03 [21.64, 27.68]	<0.001
Pregnancy order (%)			0.709
<2	4,807 (65.6)	2,950 (65.2)	
≥2	2,525 (34.4)	1,380 (34.8)	
Delivery order (%)			0.098
<2	6,162 (84.0)	3,381 (85.2)	
≥2	1,176 (16.0)	589 (14.8)	
Twins (%)			0.343
Yes	191 (2.6)	91 (2.3)	
No	7,147 (97.4)	3,879 (97.7)	
Delivery mode (%)			<0.001
Vaginal delivery	3,365 (49.8)	1,735 (43.7)	
Cesarean section	3,683 (50.2)	2,235 (56.3)	
Birth weight (kg)	3.30 [3.00, 3.60]	3.40 [3.00, 3.75]	<0.001
Birth body length (cm)	50.00 [50.00, 52.00]	51.00 [50.00, 52.00]	<0.001
Infancy feeding (%)			0.245
Pure breastfeeding	4,248 (57.9)	2,279 (57.4)	
Partial breastfeeding	2,238 (30.5)	1,261 (31.8)	
Non-breastfeeding	852 (11.6)	430 (10.8)	
Breastfeeding duration (months)	13.00 [8.00, 18.00]	12.00 [8.00, 18.00]	0.038
Family-related factors			
Number of relatives with hypertension (%)			<0.001
0	3,524 (48.0)	1,689 (42.5)	
1	1,701 (23.2)	973 (24.5)	
2	1,334 (18.2)	756 (19.0)	
3	558 (7.6)	374 (9.4)	
4	221 (3.0)	178 (4.5)	
Number of relatives with diabetes (%)			<0.001
0	5,088 (69.3)	2,547 (64.2)	
1	1,682 (22.9)	1,021 (25.7)	
2	464 (6.3)	302 (7.6)	
3	79 (1.1)	74 (1.9)	
4	25 (0.3)	26 (0.7)	
Paternal education (%)			0.077
High school degree or below	3,569 (48.6)	1,875 (47.2)	
Bachelor's degree	2,584 (35.2)	1,483 (37.3)	
Master's degree or above	1,185 (16.2)	622 (15.6)	
Maternal education (%)			0.662
High school degree or below	4,067 (55.4)	2,185 (55.0)	
Bachelor's degree	2,143 (29.2)	1,149 (28.9)	
Master's degree or above	1,128 (15.4)	636 (16.0)	
Family income (RMB per year) (%)			0.103
<100,000	3,410 (46.5)	1,862 (46.9)	
100,000–300,000	3,287 (44.8)	1,807 (45.5)	
≥300,000	641 (8.7)	301 (7.6)	

Continuous data are expressed as mean (standard deviation) in normal distributions and median [interquartile range] in skewed distributions. Categorical data are expressed as count (percentage). For continuous data, the *P* value for comparison between children with central obesity and without central obesity was derived by *t*-test for normally distributed data, by rank-sum test for skewed data, and by χ^2^-test for categorical data. BMI, body mass index.

### Selection of optimal machine-learning algorithm

**Figure 1:**
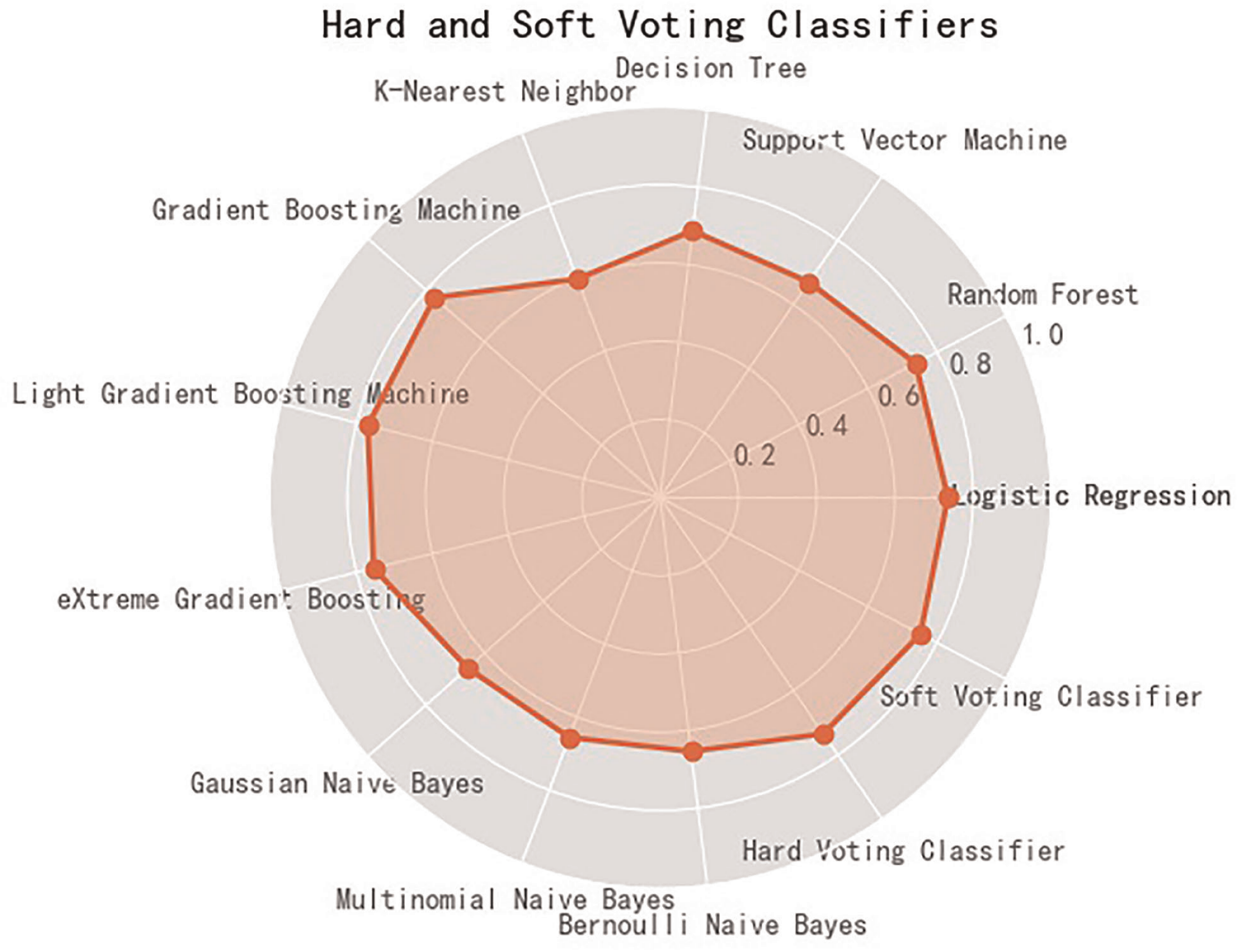
Accuracy of eleven machine learning models, along with hard-voting and soft-voting classifiers in predicting the risk of central obesity among school students. The solid red circle denotes the accuracy.

Figure 1 presents the radar-based accuracy of 11 machine-learning models, along with hard-voting and soft-voting classifiers, and detailed assessment of model performance is displayed in Table 2.

**Table 2:** Prediction performance of 11 machine-learning models for central obesity using accuracy, precision, recall, F1 score, and area under the receiver operating characteristic curve (AUROC).

Models	Accuracy	Precision	Recall	F1	AUROC
Logistic regression	0.736	0.702	0.411	0.518	0.801
Decision tree	0.687	0.547	0.560	0.554	0.657
Support vector machine	0.712	0.681	0.313	0.429	0.781
Random forest	0.738	0.702	0.421	0.526	0.784
K-nearest neighbor	0.632	0.395	0.115	0.178	0.532
Gradient boosting machine	0.771	0.700	0.591	0.641	0.828
Extreme gradient boosting	0.755	0.662	0.593	0.626	0.717
Light gradient boosting machine	0.770	0.688	0.612	0.648	0.825
Gaussian naive Bayes	0.658	0.507	0.376	0.432	0.626
Multinomial naive Bayes	0.656	0.518	0.073	0.127	0.601
Bernoulli naive Bayes	0.654	0.500	0.022	0.042	0.549

After comparison, LGBM outperformed the other 10 models. The accuracy, precision, recall, F1 score, AUROC of LGBM were 0.769982, 0.688312, 0.612323, 0.648098, and 0.825352, respectively. Importantly, the accuracy of LGBM was comparable with that of hard-voting and soft-voting classifiers. Hence in this study, LGBM was identified as the overall best machine-learning model to predict central obesity in students aged 6 to 14 years.

### Importance assessment and ascertainment of minimal set

**Figure 2:**
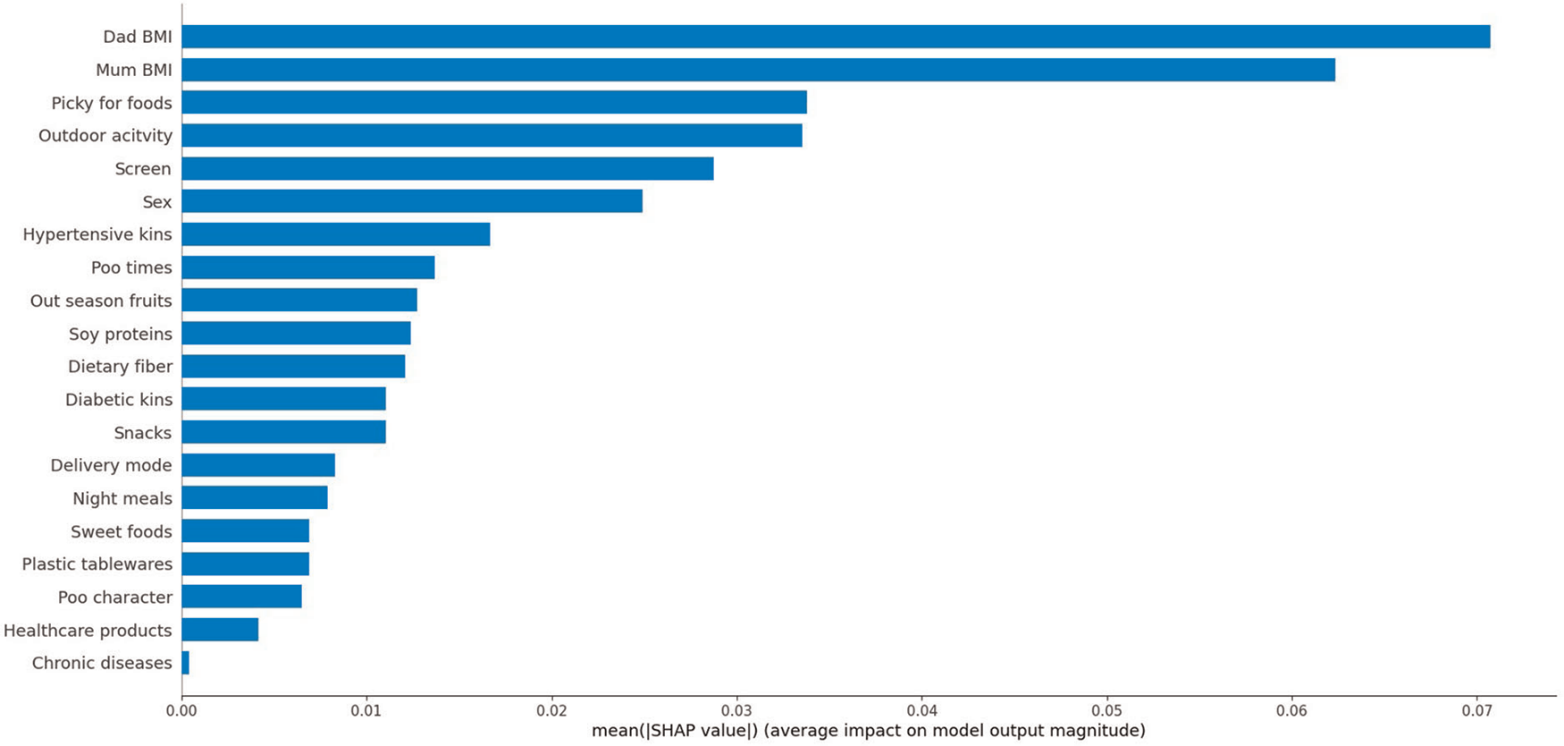
The ranking importance of top 20 factors associated with central obesity among school students.

Under the LGMB model, the importance of all studied variables was calculated and that of the top 20 variables is illustrated in Figure 2. The cumulative performance of top ten variables is shown in Table 3. After a comprehensive evaluation, the minimal set involving top 6 important variables that can predict central obesity with descent performance was ascertained, including father's BMI, mother's BMI, picky for foods, outdoor activity, screen, and sex.

**Table 3:** The distributions of areas under the receiver operating curve (AUROC), accuracy, and precision with the cumulating number of top ten important factors associated with central obesity among school students.

Number of top ten factors	AUROC	Accuracy	Precision
1	0.665	0.693	0.618
2	0.651	0.686	0.576
3	0.651	0.686	0.577
4	0.657	0.685	0.577
5	0.669	0.685	0.585
6	0.673	0.689	0.599
7	0.671	0.683	0.574
8	0.672	0.684	0.576
9	0.671	0.687	0.583
10	0.680	0.693	0.600

### Validation of minimal set

To validate whether variables in the minimal set can adequately predict central obesity relative to the whole set of variables involved, a deep-learning sequential model was adopted in both training set and validation set. As shown in Table 4, prediction performance between variables in the minimal set and in the whole set was comparable. For instance, using the optimizer Adam, model accuracy and model loss of the whole set were 67.07% and 20.28%, and that of the minimal set were 66.41% and 23.28% in the validation group.

**Table 4:** Model loss and model accuracy estimates of deep learning sequential model in both training group and validation group.

Optimizers	Training group		Validation group	
	Loss	Accuracy	Loss	Accuracy
All factors involved				
Adam	17.73%	68.20%	20.28%	67.07%
RMSPROP	18.30%	68.51%	21.69%	67.11%
SGD	18.38%	67.35%	22.55%	67.83%
6 best factors identified				
Adam	20.22%	66.92%	23.28%	66.41%
RMSPROP	21.24%	66.99%	26.17%	66.56%
SGD	23.75%	65.92%	26.40%	66.41%

Adam, adaptive moment estimation; RMSPROP, root mean square prop; SGD, stochastic gradient descent.

## Discussion

As an extension of our previous studies on general obesity and using traditional statistical models (20–23), we in this large survey, sought to identify factors in significant association with central obesity in students 6–14 years of age by use of artificial intelligence techniques. Importantly, we have identified and validated a minimal set of six important factors that can decently predict the risk of central obesity when using the optimal LGBM model relative to the whole set. The six factors are linked to central obesity of both parents, sex, and lifestyle behaviors of students. To our knowledge, this is the first study that has interrogated the risk profiles of central obesity in Chinese students in the literature.

As childhood obesity is established as a risk factor for a variety of adverse consequences in adulthood, it is of public health importance to propose an effective prediction tool for obesity and identify at-risk people at young age who might benefit from targeted interventions. Multiple prediction tools have been developed to predict obesity in children and adolescents; however, a lack of consistent reproducibility of these tools highlights the difficulties in identification of contributing factors and selection of proper models. Currently, the majority of prediction tools are developed by directly adopting linear (for continuous outcome) and Logistic (for categorical outcome) regression models, and these models cannot fully account for the collinearity and interaction of various factors. Bearing this in mind, we here adopted the advanced machine-learning and deep-learning techniques to solve these difficulties. These advanced techniques have been widely used in the medical field, especially for image recognition (24) and predicting/diagnosing diseases (25, 26).

It is widely recognized that obesity is a multifactorial disease, to which inherited and non-inherited factors contributed interactively. With the rapid economic growth and the global threat of COVID-19 pandemic, lifestyle-related behaviors have dramatically changed. For example, online education is not uncommon in modern life, and screen time in school children is related to obesity, physical activity, dry eyes, and learning ability (27). To this point, we have taken both conventional and modern lifestyle-related behaviors into consideration to identify factors associated with the risk of central obesity, a more pertinent marker than general obesity.

In this survey, the prevalence of central obesity in students from primary and junior schools was 35.1%, consistent with that of previous studies (14, 28). After a wide coverage of potential factors and the adoption of multiple machine-learning models, six important factors, including father's BMI, mother's BMI, picky for foods, outdoor activity, screen, and sex, under the LGBM model can soundly predict the risk of central obesity in school students, with performance parallel to the modeling of all factors involved. The contribution of individual factor identified to the development of central obesity is easily understandable. Taking obesity of both parents as an example, it is generally believed that obesity is “contagious”, as there is evidence that a child with one parent who is obesity is three-time more likely to become obese as an adult, while when a child's parents are both affected by obese, this child has a 10-fold risk of future obesity (29). On the other hand, family-based lifestyles in terms of dietary habits or outdoor activities, can also support the relation between obesity in parents and in offsprings (30). The contribution of individual factors to central obesity is easy to understand; however, how they act in the optimal LGBM model is elusive. Most machine-learning models (such as LGBM) are less transparent than others (such as decision tree), and their results are harder to interpret. Therefore, a high standard of transparency is required to allow parents and healthcare professionals to make informed decisions.

Some limitations should be acknowledged for this study. First, this survey is cross-sectional in nature, and so the cause-and-effect relationship between identified factors and central obesity cannot be addressed. Second, only students from a suburban district of Beijing were surveyed, and whether our findings can be extrapolated to other regions is an open question. Third, data were collected *via* online questionnaires, and recall bias cannot be totally ruled out, albeit strict quality control was made. Fourth, the findings of this study were only internally validated, and external validation in other independent groups is warranted.

Taken together, we have identified and validated a minimal set of six important factors that can decently predict the risk of central obesity when using the optimal LGBM model relative to the whole set. For the sake of clinical application, we expect that this study will not be just an end of research, but tread a path to the adoption of advanced artificial intelligence techniques in more clinical and epidemiological settings in the future.

## Data Availability

The raw data supporting the conclusions of this article will be made available by the authors, without undue reservation.
